# Tom Is Not More Likely to Imitate Lisa Than Ying: The Influence of a Model’s Race Indicated by Physical Appearance on Children’s Imitation

**DOI:** 10.3389/fpsyg.2016.00972

**Published:** 2016-06-28

**Authors:** Andrea A. R. Krieger, Corina Möller, Norbert Zmyj, Gisa Aschersleben

**Affiliations:** ^1^Developmental Psychology Unit, Saarland University, SaarbrückenGermany; ^2^Developmental Psychology Unit, Technical University of Dortmund, DortmundGermany

**Keywords:** in-group bias, imitation, children, repetition, presentation mode, culture, race

## Abstract

Recent research has shown that infants and young children up to the age of 5 years are more likely to imitate in-group members than out-group members. Cues like gender, race, age, and language are robust indicators for social categories and, thus, for group membership. Concerning imitation, research mainly focuses on language and accent, whereas race indicated by physical appearance is rarely investigated. Research has shown that the aforementioned factors served as indicators of group membership and influenced children’s imitative behavior in such ways that the in-group member was more likely to be imitated. Nevertheless, the question arises how physical appearance of a person itself influences the imitative behavior. In this study, we investigated the effect of group membership (in-group vs. out-group) in 4-year-old children (*N* = 48) on children’s imitative behavior. Children observed either an in-group or an out-group model (German vs. Chinese), defined by physical appearance only, which presented novel manual actions in four different tasks. After each presentation, children got the opportunity to imitate the target actions. Furthermore, children were either assigned to a live or a video condition to control for the influence of the presentation mode. Results indicated that 4-year-old children did not imitate the in-group model more often than the out-group model. Furthermore, there was no difference between the two presentation modes. Results were discussed on the basis of research on the in-group-out-group effect. We suggested that a pure difference in the model’s physical appearance might not be sufficient to elicit an in-group-out-group effect.

## Introduction

Adults differentiate between individuals who belong to their own group (i.e., people of the same race) and individuals who belong to a different group (i.e., people of another race). Many factors can lead to the awareness of group membership, for example gender, race, age, or language (Kinzler et al., 2010). As a consequence, social interaction between individuals is influenced by this discrimination in such ways that either benevolent behavior (i.e., helping each other), or malevolent behavior (i.e., social isolation) can occur (Fiske, 1992; Ruys et al., 2007; Trötschel et al., 2010). Moreover, research has shown decrements in out-group face recognition in adults as well as in children (Meissner and Brigham, 2001; Shutts and Kinzler, 2007). Thus, it is important to learn more about the origins of this effect and to investigate the differentiation between in-group and out-group members in children. Previous research has shown that children are able to differentiate between in-group and out-group members when membership is indicated by language. For example, 5- to 6-year-old children with another geographic origin (Northern vs. southern American) were chosen to be friends with only when they had the same accent as the participants (Kinzler et al., 2009; Kinzler and DeJesus, 2013). When asking children, which characteristic of a person is more stable, race or language, 9- to 10-year-old children name race as a stable characteristic over time, however, 5- to 6-year-old children state, that people cannot change the language they speak (Kinzler and Dautel, 2012). This indicates the important role of language in relation to race in preschoolers.

Even infants are able to differentiate between in-group and out-group members. Already by the age of 3 months, infants not only preferred faces of their own race over faces of another race, but also showed an improved recognition of faces of their own race (Sangrigoli and De Schonen, 2004; Bar-Haim et al., 2006). Furthermore, 10-months-old infants selectively preferred toys that were offered by someone with a native accent than from someone with a foreign accent (Kinzler et al., 2007). Similarly, 14-month-old infants imitated actions of a model who spoke their native language more often than actions of a model who spoke a foreign language (Buttelmann et al., 2013). Thus, there is ample evidence that language is an important factor influencing the in-group-out-group effect already in infancy.

However, it is still unclear whether children show selectivity in their behavior when language is not available as a cue for group membership, for example, when only the physical appearance of a model is available. Aboud’s (1988) sociocognitive theory claims that two overlapping sequences of perceptual-cognitive development influence children’s attitude toward other groups. One sequence of development is concerned with changes in the child’s focus of attention, in which young children focus on their own beliefs and older children focus on categories of other people as well (Aboud, 1988). Thus, younger children’s opinion is influenced by their own awareness of characteristics of other people, whereas preferences of older children are built because characteristics of group membership are influenced by the attitude toward other people. The other sequence involves an affective-perceptual process, which includes attachment to familiar people or objects and fear toward the unknown and is determined by physical appearance up to the age of 7 years. Applying the affective-perceptual process to imitation studies, children should approach the in-group model and imitate more of her actions as compared to the out-group model, even if group membership is only indicated by physical appearance. There are only few studies investigating the influence of race indicated by physical appearance on imitational behavior. Shutts et al. (2010) showed faces of unfamiliar children differing among others in race. Faces were coupled with voice records indicating the name and the preferences of each specific child. 3-year-old children were asked to choose between objects and activities endorsed by those faces. No effect of race was found. The authors concluded that race is not encoded spontaneously and, thus, does not influence children’s preferences and choices at this age (Shutts et al., 2010). In another study, 5-year-olds either saw three members of an out-group or of a neutral group demonstrating the same action. Group membership was defined by differently colored scarfs. After having seen the out-group members, children produced more contrasting actions than actions, which matched those of the out-group, whereas children’s actions matched those of the neutral group after having seen those (Oostenbroek and Over, 2015). However, this effect was only shown for 5-year-old but not for 4-year-old children. Furthermore, Diesendruck and HaLevi (2006) showed an advantage for labels over physical appearance in 5-year-old children. However, they used line drawn faces instead of pictures of real people and the experimenter told participants, which group they were in (Diesendruck and HaLevi, 2006). In accordance with the aforementioned findings, where physical appearance was defined through colors or drawings more research concerning the physical appearance of a living person and its influence on the imitative behavior of children is needed.

Imitative behavior is not only influenced by a model’s group membership but also by the presentation mode. When infants observe an action on TV, they imitate less action steps as compared to when they observed a real-life model (Barr and Hayne, 1999). This so-called *video deficit effect* (Anderson and Pempek, 2005) has been documented in a variety of studies showing that up to the age of 3 years children imitated more actions in live presentation than in video presentation conditions. Thus, from 3 years onward children seem to learn from video and live presentations likewise (Troseth and DeLoache, 1998; Troseth et al., 2006; Howard et al., 2015). However, Reiß et al. (2014) showed that 4-year-old children performed reliably better in the live than in the video condition when Theory of Mind tasks were presented. Based on these somehow inconsistent results, we decided to control for possible distracting influence of the video presentation, and thus a live model condition was introduced in the present study as well.

The aim of the present study was to investigate whether group membership indicated by physical appearance of the model influences 4-year-olds’ imitative behavior. To this end, we constructed four novel tasks with different three-step actions. Both a Chinese and a German model presented these actions, and children were given the opportunity to imitate these actions immediately afterward. While these actions were presented on video, we also introduced a live condition (German model only) to control for the possible distracting influence of the video presentation. In the video condition, German preschoolers observed a German and a Chinese model that presented novel, manual actions on objects in two runs. In the first run, children were presented either with the German or with the Chinese model that demonstrated the actions. In the second run, children were presented with the other model that presented the same actions again. After each action, children were allowed to play with the objects. In the live condition, children observed only the German model in both the first and the second run.

We expected children to imitate the in-group model more likely and more quickly than the out-group model if physical appearance is sufficient. There is clear evidence for 5-year-old children to prefer in-group models over out-group models (Diesendruck and HaLevi, 2006; Oostenbroek and Over, 2015), however, results concerning 3- to 4-year-old children are less clear (Diesendruck and HaLevi, 2006; Shutts et al., 2010). Awareness of race in children begins to emerge at the age of 3 years (Nesdale, 2001), therefore, we tested 4-year-old children in the current study to guarantee that race is detected by the children. Moreover, we expected no difference in imitation performance between video and live presentation at this age.

## Materials and Methods

### Participants

The final sample consisted of 48 German children (*M* = 4;5 (years; months); range = 3;9-5;0). Additional four children were tested but not included in the final sample due to procedural errors. Children were randomly assigned to two experimental groups (live presentation, *N* = 24; video presentation, *N* = 24). Parents were recruited by telephone from a list of families who had earlier expressed interest in volunteering for research on child development. They received a recompense for travel expenses and children were given a small gift and a certificate for participating. This study has been conducted in accordance with ethical guidelines and received ethical clearance by the local ethics committee at the Saarland University.

### Materials

**FIGURE 1:**
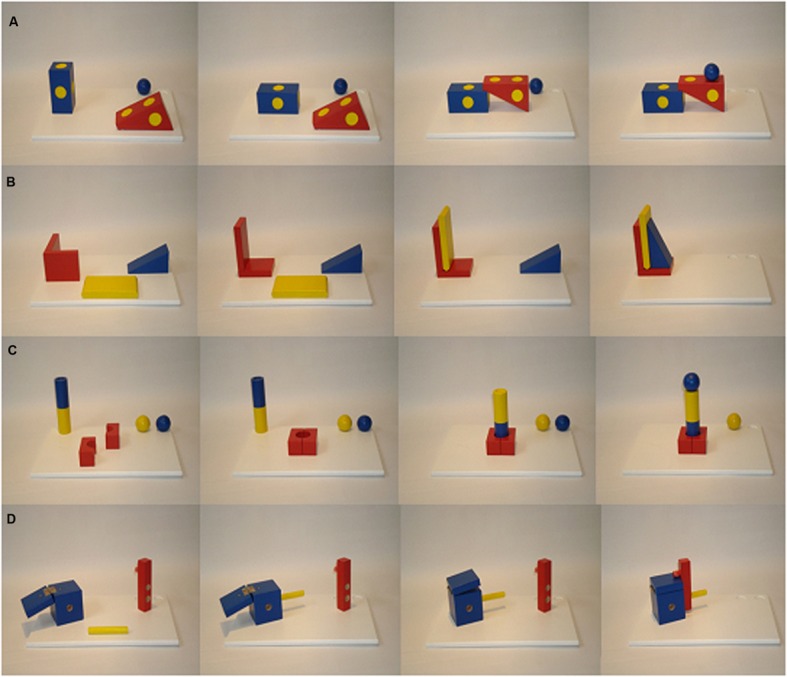
**Three-step-action sequence of the four tasks.** Starting position and the subsequent three action steps of the bridge **(A)**, the bookend **(B)**, the rod **(C)** and the box **(D)**.

There were four manual tasks. Each task consisted of three wooden building bricks, which were purpose-built (see **Figure 1**). The first task, named *the bridge*, consisted of one blue block [9 cm (length) × 4.5 cm (width) × 4.5 cm (height)], one red rectangular prism (6 × 10 × 4.5 cm) and one blue ball (diameter = 3.3 cm). The red prism and the blue block had yellow millings on each side. The second task, called *the bookend*, consisted of a red L-shaped object (6 × 7 × 10.5 cm), a yellow flat building brick (1.5 × 11.5 × 5.9 cm) and a blue rectangular prism (4.5 × 9 × 4.5 cm). The third task, named *the rod*, was made up of a rod colored half blue and half yellow (length = 11.6 cm; diameter = 3.2 cm) and two balls of different color (blue/yellow; diameter = 3.3 cm). Additionally, there was a red squared block (6 × 7.6 × 6 cm) consisting of two brick-formed identical parts, which were hold together by a magnet. In the middle of the squared side of the block there was hole (diameter = 1.4). The fourth task, called *box*, contained of a blue box (7.3 × 6 × 6 cm), a yellow stick (8 cm; diameter = 1.2 cm), and a red bar (10 × 2.2 × 2.2 cm) with a nub (diameter = 1.5 cm) and two holes under the nub (diameter = 1.3 cm). The blue box had six holes in the side walls (diameter = 1.6 cm) and a flap, which was provided with repelling magnets. Thus, a bit pressure was needed to close it.

For the *bridge*, the model tipped over the blue block on its left side. Then, one edge of the red rectangular prism was placed on one edge of the blue block. Finally, the blue ball was placed on one of the upper yellow millings (see **Figure 1**). For the *bookend*, the model put the L-shaped red object in an upright position. Then, the yellow flat building brick was leaned on the longer side of the L-shaped object. Finally, the blue rectangular prism was leaned on the yellow flat building brick with the longer side of the right angle. For the *rod*, the model put together the two parts of the red squared block with the round opening. Then, she rotated the rod with a 180° turn and positioned it within the round opening of the red squared block. Finally, the blue ball was positioned on top of the rod. For the *box*, the first step was to put the yellow stick into the opening of the box, which the model was facing directly. Then, the model closed the box, which flapped because of the repelling magnets. Finally, she pushed the red bar on the yellow stick and used it to close the lid of the box.

**FIGURE 2:**
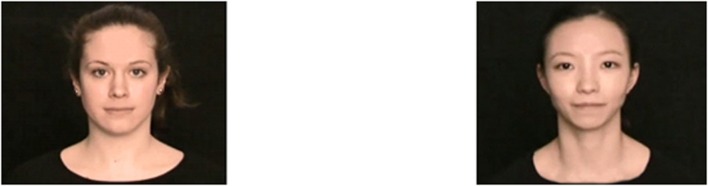
**The German Model and the Chinese Model.** Photographs of the German (left) and the Chinese (right) model.

Two female adult models with different race (Chinese vs. German) demonstrated the manual tasks (see **Figure 2**). Both models were comparable in terms of age (31 years vs. 25 years), hair and eye color, but differed in race-specific features (facial proportions and eye relief). Both the Chinese model and the German model were shown in the video condition, and the same German model modeled the actions in the live condition. In two prestudies, one with students, one with children, we checked whether the models differed in other features than their physical appearance. When students (*N* = 59) rated several characteristics of the models (e.g., sympathy), no difference was obtained except that the German model was rated more sociable than the Chinese model. 4-year-old children (*N* = 17) answered questions about sympathy and were asked, which characteristics the models have in common (e.g., openness toward other people, self-confidence). No significant differences between the two models were obtained.

### Design

There were two runs, each consisting of the presentation of the four different tasks being presented in counterbalanced order across participants. The German model presented the tasks in the live condition, whereas in the video condition the tasks were presented by the same German and the Chinese model, one run with the German and one run with the Chinese model. The order of the models was counterbalanced across participants. After each task, children were given the possibility to play with the objects. Thus, the influence of the model’s race (Chinese vs. German model) was tested in a within-subject design in the video condition. The influence of the presentation mode was tested in a between-subject design (live vs. video; German model). To check the pure factor repetition without an influence of the models race, we analyzed this factor in the live condition (German model only; 1st vs. 2nd run).

### Procedure

Children sat on a high chair at a table (74 × 103 × 82 cm) in front of a blue covered wall with an opening (60 cm length) in the middle of it, comparable to a “puppet theater.” The opening could be closed by a curtain. In the live condition, children saw the German model performing the manual actions through this opening. In the video condition, a monitor (24′′, 50/60 Hz) was positioned exactly into the opening. All aspects of the live demonstration were closely matched to the video demonstration (i.e., the velocity and amplitude of the actions, the duration of the demonstration). If the child looked away from the model, the experimenter who was standing on the side during the presentations reminded the child to look back to the model and focused the child’s attention back to the demonstration. Both the video and the live condition followed the same general procedure. An experimenter welcomed the parent and the child. While the parent waited in an extra room and filled in questionnaires concerning some background information of the child (e.g., age, noticeable problems) the child was led to a separate room and the experimenter instructed the child (“Soon you will see a friend of mine, who is playing with different toys”). First, a bell rang in order to draw the children’s attention to the closed curtain. Then, the curtain opened and the model looked directly at the child for 4–5 s. Then, the model looked at the first object and performed the manual action with it. After performing the three steps each task consisted of, the model looked toward the child again. Then, the curtain closed again. Note, that the model did not talk, thus, no language was involved. The experimenter gave the identical objects to the child with a neutral instruction (“Now it is your turn to play with the toys!”). Children were allowed to play with the objects for 30 s, starting when the child touched the first object. The child was told to ring the bell, which was positioned next to it, whenever she/he finished playing with the objects. The experimenter removed the objects after 30 s or after the child rang the bell, and the presentation of the next task started. After the first run, the second run started immediately without a delay in between. When children had completed both runs, they could choose a toy as a reward and were then brought back to their parents. Each session was videotaped by a camera (Canon Legria FS200E) directed frontally at the child, and a second camera (Canon Legria FS406) recorded the child and the model from behind.

### Coding and Analyses

Children’s behavior was coded from the videotapes. First, latency was coded as the time between the time when the experimenter had placed the objects in front of the child and the child’s first touch of an object. Additionally, we coded the number of imitated steps. A step was coded as imitated when children performed the same movement with the same object as the model had demonstrated at any point during the response period. Children could receive a score from 0 to 3 in every single task leading to a sum score ranging from 0 to 12 for each run. These two dependent variables (number of imitated steps and latency of first touch) were taken in account for the main hypothesis, as well as for the control hypotheses. Furthermore, we coded the time children spent looking at the video and the live presentation to check for any differences of children’s attention. No significant difference could be found concerning looking time (Wilcoxon text: *z* = -1.48; *p* = 0.138). 60% of the videos were coded by a second independent rater. Interrater agreement was *κ* = 0.81, *p* < 0.001.

## Results

### In-group-out-group Effect

In order to investigate whether there were differences between the two models concerning latency and number of imitated steps a dependent-sample *t*-test was calculated. Results revealed that children did not imitate more action steps when observing the in-group model (*M* = 9.25; *SD* = 3.03) compared to the out-group model (*M* = 9.54; *SD* = 2.65), *t*(23) = 0.71; *p* = 0.484. Similar results were found for latency. Children did not start to play faster with the objects after having watched the German model performing the action as compared to the Chinese model, *t*(23) = -1.62; *p* = 0.119. To control for order effects, two independent-sample *t*-tests were calculated. No effect of order (Chinese-German vs. German-Chinese) was obtained, not for the number of imitated steps, *t*(11) = -1.30; *p* = 0.220 and not for latency, *t*(11) = -0.56; *p* = 0.587.

### Presentation Mode

Children’s imitation performance did not differ as a function of the presentation mode (German model only). No significant difference was found for the number of imitated steps (live: *M* = 8.38; *SD* = 3.32; video: *M* = 9.54; *SD* = 2.65; *t*(46) = -1.17, *p* = 0.185) as well as for the latency (live: *M* = 7.67; *SD* = 5.28; video: *M* = 7.20; *SD* = 4.49, *t*(45) = 0.46; *p* = 0.748).

### Repetition Effect

**FIGURE 3:**
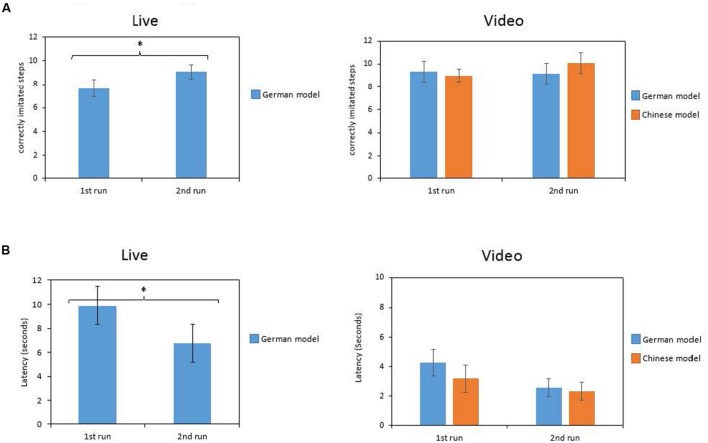
**Number of correctly imitated steps **(A)** and latency **(B)** in the live and in the video presentation depending on the run (1st and 2nd) and on the race of the model (German vs. Chinese; video presentation only).** *p < 0.05.

To test the repetition effect, paired *t*-tests comparing the values obtained in the first and the second run in the live condition were computed (German model only). There were significant differences in the number of imitated steps [*t*(23) = -3.29, *p* = 0.003; see **Figure 3A** left part]. Children copied fewer steps in the first run (*M* = 7.67; *SD* = 3.45) compared to the second run (*M* = 9.08; *SD* = 2.99). Furthermore, mean latency also differed significantly [*t*(21) = 2.32, *p* = 0.030]. In the first run, children started to play later with the objects than in the second run (1st: *M* = 9.90; *SD* = 5.45; 2nd: *M* = 6.77; *SD* = 5.52; see **Figure 3B** left part).

Furthermore, two repeated-measures variance analyses with the within-factor model (German vs. Chinese) and the within-factor repetition (1st vs. 2nd run) for correctly imitated steps and latency were calculated to control for repetition effects in the video condition. Results revealed, that for correctly imitated steps there were no significant main effects for repetition [*F*(1,11) = 0.60; *p* = 0.455] and for model [*F*(1,11) = 1.10; *p* = 0.317] as well as there was no significant interaction between repetition and model, *F*(1,11) = 0.63; *p* = 0.630 (see **Figure 3A** right part). For latency, there was a significant main effect for repetition, *F*(1,11) = 5.53; *p* = 0.038. Children started faster playing with the objects during the second run (*M* = 2.44; *SD* = 1.59) compared to the first run (*M* = 3.74; *SD* = 2.69; see **Figure 3B** right part). There was no main effect for model [*F*(1,11) = 3.11; *p* = 0.106] and no significant interaction between repetition and model, *F*(1,11) = 0.313; *p* = 0.587.

Finally, to compare the live and the video condition two mixed ANOVAs with the within-factor repetition (1st and 2nd run) and the between-factor presentation mode (live vs. video) were calculated. Concerning the number of imitated steps, results revealed a significant main effect for repetition with *F*(1,46) = 11.40; *p* = 0.002, no significant effect of the presentation mode, *F*(1,46) = 1.47; *p* = 0.232, and no significant interaction between repetition and presentation mode, *F*(1,46) = 1.98; *p* = 0.166. Similarly, for the latencies, results revealed a significant main effect for repetition with *F*(1,46) = 9.46; *p* = 0.004, no significant effect of the presentation mode, *F*(1,46) = 0,04; *p* = 0.834, and no significant interaction between repetition and presentation mode, *F*(1,46) = 0,37; *p* = 0.544.

## Discussion

The aim of the present study was to investigate the influence of the model’s group membership indicated by physical appearance on 4-year-olds’ imitative behavior. The results showed that children did not imitate more action steps after having observed the German model compared to the Chinese model. Similarly, they did not differ in latency to the first touch. At first view, this is not in line with prior research showing that children take the models’ group membership into account when they imitate others (e.g., Diesendruck and HaLevi, 2006; Buttelmann et al., 2013; Howard et al., 2015). However, these studies used other indicators than physical appearance as a cue for group membership. We will discuss possible explanations below.

As expected, there were no differences between live and video presentation concerning the imitation performance of the children. This result conformed to prior research, which showed that the video deficit occurs up to the age of three years (e.g., Barr, 2010; but see Reiß et al., 2014 for conflicting results). The lack of this effect also cannot be attributed to differences in the details of the live and video demonstration as the models were well trained to act standardized. Furthermore, we arranged the context of the videos in the same way as it was during the live condition. Previous studies found the in-group-out-group effect when using televised models between 1 and 3 years of age (Buttelmann et al., 2013; Howard et al., 2015). Thus, we believe that it is unlikely the video presentation mode obscured an in-group-out-group effect in children concerning the model’s race. Future research might test this assumption in a full-factorial design with video and live presentations for the in-group and out-group model.

Finally, children imitated more steps after the second run than after the first run and started playing faster with the objects. The latter result was found in both the live presentation and the video presentation. This indicates that perhaps 4-year-old children benefit from multiple runs because they get more comfortable with the objects and thus started faster playing with the objects. We assume that this improvement might be due to a higher familiarization to the actions. Concerning the number of imitated steps, there was only a significant improvement within the live condition, where children had only seen the German model presenting the actions. Although the corresponding analysis of variance revealed a significant main effect of repetition and no significant interaction of presentation mode and repetition, this effect can mainly be ascribed to the live condition as indicated by the mean values (see **Figure 3A**). It might well be, that the more children saw the actions, the better they could understand them and thus imitate them more frequently (Sell et al., 1995). Furthermore, children had to focus on the actions twice which could lead to a better rehearsal and storage of the actions (Bandura, 1986). Thus, their recollection was better and they were more likely to imitate. However, we have to take into account that the improvement in imitative behavior was greater after having seen the live model as compared to the video presentation. In fact, the starting level of imitated steps was lower in the live condition compared to the video presentation and only reached the same level in the second run. Thus, it might be that in the live condition children had to acclimatize to the environment first as it was somehow artificial because the model did not communicate with the child at all.

Concerning the in-group-out-group effect, there are different possible explanations for why the finding is in contrast to prior findings. First, we have to consider the possibility that the results are due to an artefact of the tasks. The imitation tasks could have been too easy for children and thus were performed independently of the model. To control for ceiling effects, we looked at the total amount of children imitating all of the possible 12 steps correctly. Only 5 children (10%) were able to imitate all steps correctly in the first as well as in the second run. This indicates that the tasks were not too easy.

Second, the age of the children could be responsible for these diverging results. Various studies showed that by the age of 5 but not 4 years children take the race into account when dealing with imitation and drawing inferences (Hirschfeld and Gelman, 1997; Oostenbroek and Over, 2015). Similarly, at the age of 3 years children do not seem to comprise the race to guide their behavior or their preferences. For example, Shutts et al. (2010) found, that 3- to 4-year-old children did not use racial information of the models to guide their own preferences for novel items. Furthermore, Kircher and Furby (1971) did not find evidence for 3-year-old children but for 4-year-old children to use race-based information to build preferences. For infants, research also found evidence that there is a preference toward the in-group, which influenced, for example, the eye movements in 3- and 10-month-old infants (Sangrigoli and De Schonen, 2004; Bar-Haim et al., 2006). However, the latter ones were looking time studies and thus can only be compared to results obtained in imitation studies to a very limited amount. Concerning preschool children, there might be a developmental process concerning the awareness of differences between groups and the active use of this information for decisions, like preferences and imitational behavior. Whereas 5-year-old children seem to take into account the race of the model, 3-year-olds do not. Concerning the age of 4 years, findings reported in the literature are less clear. Thus, the age of the children might be one reason for the fact, that we did not find evidence for an in-group-out-group effect in the present study.

Another possible explanation is that the model’s mere physical appearance is not sufficient to influence 4-year-old’s imitative behavior. Studies investigating how children draw inferences about psychological properties found that children did not use physical appearance but verbal labels about the models’ race (Diesendruck and HaLevi, 2006). Most studies, which investigated in-group-out-group effects on imitation, used language as a marker for group membership (Kinzler et al., 2007, 2011; Buttelmann et al., 2013; Howard et al., 2015). In contrast, we neither used any labels for the models nor did the models speak a word during the sessions. When physical appearance is used to indicate a model’s racial group membership, it might not be salient enough to serve as a cue for group membership (Diesendruck and HaLevi, 2006). In line with this argument, Shutts et al. (2010) observed that a model’s age and gender is more important than a model’s race when 3-year-olds choose between objects, which were presented by models differing in age, gender and race. In the present study, we kept the models’ age and gender constant in order to analyze the genuine effect of physical appearance. Furthermore, the familiarity of Chinese people could be one possible reason why physical appearance was not salient enough to influence children’s imitative behavior. Perhaps children are familiar with the physical appearance of Chinese people because they are around them in everyday life.

In sum, it might well be that the influence of the model’s race on children at different ages is moderated by language. That is, language might offer more salient information about the model’s race than physical appearance. Thus, the role of language and, most importantly, their interplay should be analyzed in more detail in further studies. Furthermore, the age of children should be analyzed in more detailed studies, which could include also children of the age of 3 and 5 years.

## Conclusion

Various studies found evidence that children’s imitational behavior is influenced by group membership. Belonging to a group is communicated through features like a model’s age, gender, and language. The current study manipulating physical appearance only did not show evidence that the model’s race elicited the in-group-out-group effect in 4-year-olds. We propose that additional information especially language (e.g., labels) is necessary to highlight group membership at this age and to result in group-specific imitative behavior in children.

## Author Contributions

All authors originated the idea for the study and contributed to the development of the conceptual framing, and the analysis framework. AK was the primary conductor of analyses and wrote the most of the manuscript. GA, NZ, and CM revised and reworked the manuscript. GA wrote some section and made suggestions for additional analyses.

## Conflict of Interest Statement

The authors declare that the research was conducted in the absence of any commercial or financial relationships that could be construed as a potential conflict of interest.
